# Identification of novel *MITF* mutations in Chinese families with Waardenburg syndrome type II

**DOI:** 10.1002/mgg3.1770

**Published:** 2021-07-29

**Authors:** Jing Wang, Yu Lu, Xiaohong Yan, Tian Shen, Linke Li, Yufang Rao, Bo Tan, Wenyu Xiong, Jing Cheng, Yu Zhao, Huijun Yuan

**Affiliations:** ^1^ Department of Oto‐Rhino‐Laryngology West China Hospital West China Medical School Sichuan University Chengdu China; ^2^ Institute of Rare Disease West China Hospital of Sichuan University Chengdu China; ^3^ Medical Genetics Center Southwest Hospital Army Medical University Chongqing China

**Keywords:** incomplete penetrance, *MITF*, sensorineural hearing loss, Waardenburg syndrome

## Abstract

**Background:**

Waardenburg syndrome (WS) is a rare autosomal‐dominant syndrome and is characterized by sensorineural hearing loss and pigment abnormalities. It is subdivided into four types according to the clinical characteristics. *MITF* is one of the major pathogenic genes for type II. The aim of this study was to investigate *MITF* mutations and the clinical characteristics of WS type 2 (WS2) in four Chinese families.

**Method:**

Clinical diagnoses were based on detailed clinical findings. Six WS2 patients from four unrelated Chinese families were enrolled. Massively parallel DNA sequencing was used to find pathogenic genes and Sanger sequencing was used to confirm the variants detected.

**Results:**

Sensorineural hearing loss was observed in four of six patients, three had heterochromia iridis, and five have freckled faces. We identified three novel *MITF* heterozygous mutations (c.831dupC, c.650G>A, and c.711‐2A>G) and one recurrent heterozygous mutation (c.328C>T) in the four WS2 families. Intra‐familial phenotypic variability and incomplete penetrance were found in WS2 patients with pathogenic variants of *MITF*.

**Conclusion:**

Genetic diagnosis was performed for the involved four families based on the clinical manifestations. Four heterozygous mutations were identified in the *MITF* gene. Our findings expanded the phenotypic and genotypic spectrum of WS.

## Graphical abstract

MITF mutations and the clinical characteristics of WS2 in Chinese families. Four novel heterozygous mutations in the MITF gene. Intra‐familial phenotypic variability and incomplete penetrance in the MITF gene.

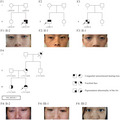

## 1 INTRODUCTION

Waardenburg syndrome (WS) is a rare autosomal‐dominant syndrome, accounting for 2–5% of the patients with congenital deafness (Waardenburg, 1951). It is characterized by sensorineural hearing loss and pigment abnormalities, including abnormal iris pigmentation, patchy depigmentation or freckled face, and a white forelock or premature graying of the hair (Song et al., 2016). WS has high phenotypic and genotypic heterogeneity because of its incomplete penetrance and diversity of pathogenic genes (Farrer et al., 1994). It is subdivided into four types according to the clinical characteristics (Chen et al., 2010). Type I (WS1; OMIM 193500) is distinguished from type II (WS2; OMIM 193510) by the presence of dystopia canthorum. Type III (WS3; OMIM 148820) is similar to type I, but is accompanied by upper limb abnormalities. Type IV (WS4; OMIM 277580) is type II associated with Hirschsprung disease. Six genes have been confirmed to be related to WS. *PAX3*(OMIM 606597) is associated with types I and III, *MITF*(OMIM 156845), *SNAI2*(OMIM 602150), and *SOX10*(OMIM 602229) are related to type II; and *EDNRB*(OMIM 131244), *EDN3*(OMIM 131242), and *SOX10* are involved in the pathogenesis of type IV (Pingault et al., 2010).

Microphthalmia‐associated transcription factor (*MITF*) is a basic helix‐loop‐helix‐leucine zipper (bHLH‐Zip) transcription factor and is a pathogenic gene in WS. *MITF* has several promoters and encodes at least seven isoforms (Vachtenheim & Borovanský, 2010). The promoters associated with each isoform contribute to their tissue‐specific expression and functions (Steingrímsson et al., 2004). The M isoform is specifically expressed in melanocytes, has nine exons, and encodes 419 amino acids (Chen et al., 2016). Most WS‐associated *MITF* mutations are in exons 7, 8, and 9, which correspond to the basic, HLH, and leucine zipper domains, respectively, and are highly conserved in vertebrates and invertebrates (Pingault et al., 2010). *MITF* also contains two transactivation domains (TAD). The mutants may impair its transcription activities, phosphorylation, DNA binding, and nuclear localization (Smith et al., 2000; Takeda et al., 2000), and affect activation of the tyrosinase (*TYR*) gene (OMIM 606933), which is involved in melanocyte differentiation (Nobukuni et al., 1996). The absence of melanocytes can cause pigment abnormalities in the skin, hair, and eyes, and affect hearing function in the inner ear.

Here, we present four Chinese families with WS2 with sensorineural hearing loss, pigmentation abnormalities of the iris, and freckled faces caused by variation in the *MITF* gene. One recurrent and three novel mutations were associated with WS2.

## 2 MATERIALS AND METHODS

### 2.1 Editorial policies and ethical considerations

This study was approved by the Ethics Committee of West China Hospital (No. 2020–606). Informed consent was obtained from all study participants.

### 2.2 Family data collection

We included probands who were diagnosed with WS2 at the Department of Otolaryngology of West China Hospital, Chengdu, China.

All patients completed a questionnaire and underwent a physical examination. Hearing was assessed by pure‐tone audiometry at frequencies of 250, 500, 1,000, 2,000, 4,000, and 8,000 Hz. The severity of hearing loss was normalized by the better side. The hearing level was classified into five grades: normal (<20 dB HL), mild (20–40 dB HL), moderate (41–70 dB HL), severe (71–95dB HL), and profound (>95 dB HL) deafness. The W index (in mm) was calculated for all of the family members as follows: *X* = (2*a* – (0.2119*c* + 3.909))/*c*, *Y* = (2*a* – (0.2479*b* + 3.909))/*b*, *W* = *X* + *Y* + *a*/*b*. Here, *a*, *b*, and *c* are the inner canthal, interpupillary, and outer canthal distances, respectively.

### 2.3 Massively parallel DNA sequencing

Genomic DNA was isolated from peripheral blood using the AxyPrep‐96 Blood Genomic DNA Kit (Axygen Biosciences) according to the manufacturer's instructions. Massively parallel DNA sequencing of 40 genes known to be associated with hearing loss was conducted in all family members. A genomic DNA library was constructed following the manufacturer's instructions, using target capture (Agilent Technologies). All exons, flanking introns, and splicing regions of the 40 genes were captured. The captured DNA fragments were sequenced on a HiSeq2000 (Illumina).

### 2.4 Bioinformatic analyses

The data were analyzed and bioinformatics data were processed following standard Illumina procedures. Raw sequence reads were mapped to the human reference genome (GRCh37/hg19) using the Burrows–Wheeler Aligner (ver. 0.7.15). Variants were called using Genomic Analysis Tool Kit best practices. Variants were annotated using Variant Effect Predictor and filtered for minor allele frequency (MAF) in gnomAD and variant consequence. PhyloP and GERP++ were used to predict conserved regions. Sorting Intolerant from Tolerant (SIFT), Polyphen‐2, LRT, MutationTaster, and Combined Annotation Dependent Depletion (CADD) were used to predict deleteriousness. The pathogenicity of the variants was analyzed following the American College of Medical Genetics and Genomics (ACMG) recommendations for the interpretation of sequence variants (Richards et al., 2015).

### 2.5 Validation by Sanger sequencing

Four pairs of primer sequences were designed for variants in the *MITF* gene (NM_000248.3). (Table S1). To confirm the detected variants, standard Sanger sequencing protocols were performed on an ABI 3500xL Dx Genetic Analyzer (Applied Biosystems; Thermo Fisher Scientific). Gene mutations were analyzed using Chromas (ver. 2.6.5).

## 3 RESULTS

### 3.1 Clinical evaluation of four Chinese families with WS2

**FIGURE 1:**
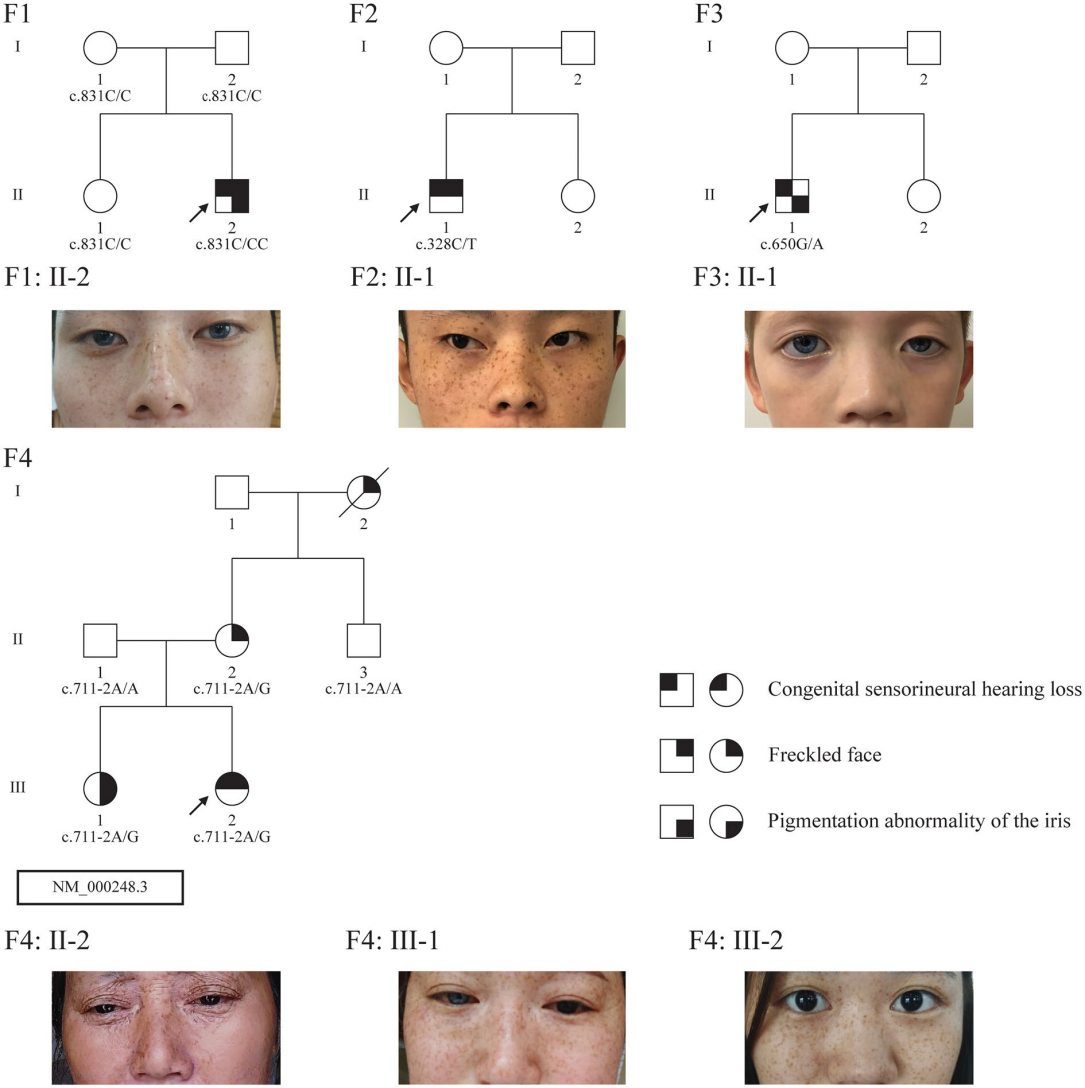
The pedigree and clinical features of involved WS2 families

**FIGURE 2:**
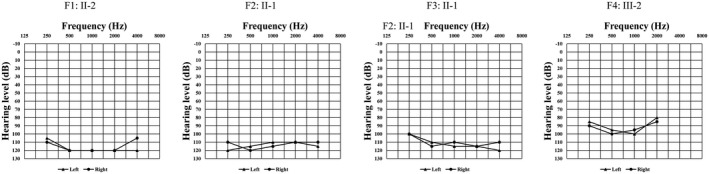
Pure tone audiometry in WS2 families

In total, six patients, including two males and six females, and some of their family members were recruited for the clinical examinations. The four families were from Sichuan Province, China, and all family members are Han Chinese. Sensorineural hearing loss was observed in four of the six patients, three patients had heterochromia iridis, and five patients had freckled faces. Figure 1 shows the patients’ pedigrees and clinical characteristics. All the patients with hearing loss were bilateral severe to profound deafness (Figure 2). No dystopia canthorum, upper limb abnormalities, or Hirschsprung disease was observed in any subject (Table 1).

**TABLE 1:** Clinical and molecular features of individuals of the WS family

Subject	F1:II‐2	F2:II‐1	F3:II‐1	F4:II‐2	F4:III‐1	F4:III‐2
Age	22	17	9	52	30	20
Sex	M	M	M	F	F	F
Sensorineural hearing loss	+	+	+	−	−	+
Heterochromia iridis	+	−	+	−	+	−
Freckled face	+	+	−	+	+	+
*MITF* mutation (het)	c.831dupC	c.328C>T	c.650G>A	c.711‐2A>G	c.711‐2A>G	c.711‐2A>G
Protein change	p.Asn278Glnfs*12	p.Arg110Ter	p.Arg217Lys	−	−	−
Reference	This study	Bocángel et al. (2018)	This study	This study	This study	This study

### 3.2 Identification and verification of pathogenic mutations

**FIGURE 3:**
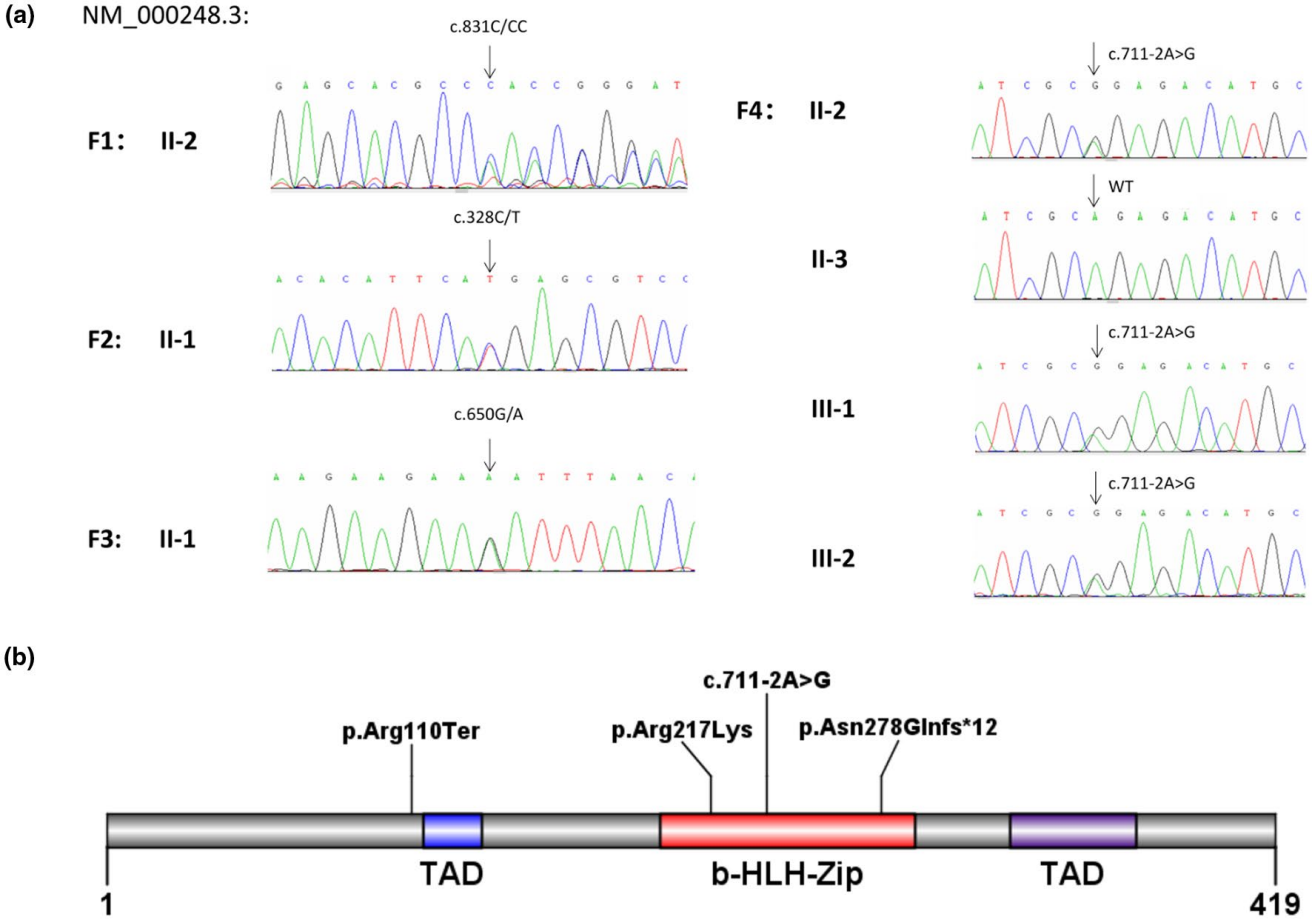
Sanger sequencing and the location of mutation sites in *MITF*. (a) Sanger sequencing results of the patients’ family. (b) Structure of the MITF protein and the positions of the variants

We identified four heterozygous *MITF* mutations by massively parallel DNA sequencing and Sanger sequencing (Figure 3). Heterozygous mutations were found in families 1 (II‐2; c.831dupC), 2 (II‐1; c.328C>T), 3 (II‐1; c.650G>A), and 4 (II‐2, III‐1, and III‐2; c.711‐A>G). The locations of the mutations at the protein level are shown in Figure 3. None of the variants were in the dbSNP, ExAC, 1000 Genomes Project, or gnomAD databases. The four mutations were compared to the Deafness Variant Database (http://deafnessvariationdatabase.org/) and ClinVar (https://www.ncbi.nlm.nih.gov/clinvar/). This revealed that mutation c.328C>T had been reported, while the other three are being reported here for the first time.

The novel frameshift variant c.831dupC in exon 8, causes the change of 12 amino acids after 278 amino acid, resulted in the early termination of polypeptide chain. According to ACMG/AMP variant interpretation guidelines (2018) for genetic hearing loss (Oza et al., 2018), it is a PVS1 variant. In addition, mutation c.831dupC in Family 1 was a *de novo* mutation (PS2), Therefore, it is considered to be pathogenic (PVS1+PS2).

**FIGURE 4:**
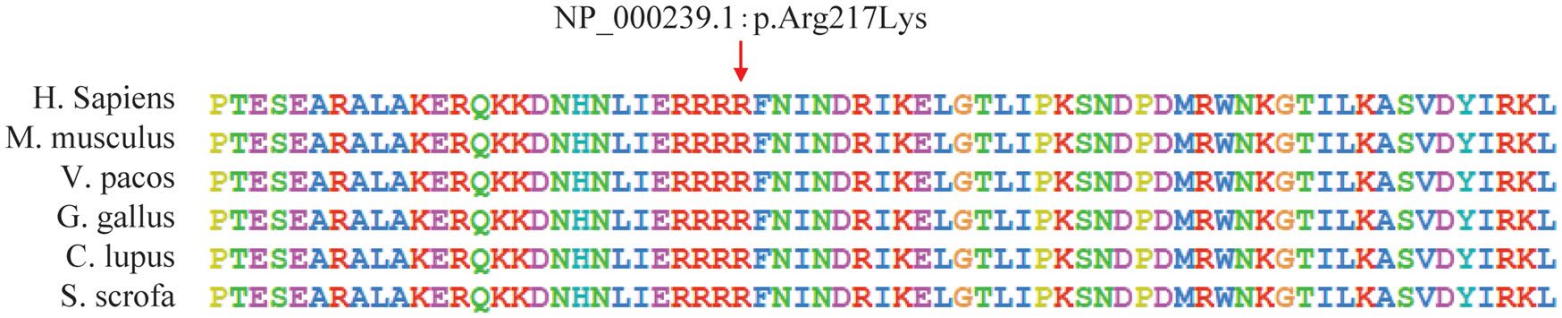
Arginine 217 of MITF is conserved in different species

The novel mutation c.650G>A is a missense variant of exon 7, causing the transformation p.Arg217Lys, a residue in the highly conserved bHLH‐Zip domain. Besides, this locus is highly conserved in many species (Figure 4). The variant is not in the dbSNP, ExAC, 1000 Genomes Project, or gnomAD databases (PM2). However, missense changes at the same codon as another pathogenic missense variant which was reported before (PM5). It was predicted to be deleterious by SIFT, Polyphen‐2, MutationTaster, and LRT. And REVEL score ≥0.7 (PP3). In addition, it had a CADD score of 31. Patient's phenotype is highly specific for *MITF* (PP4). In conclusion, the variant is considered to be likely pathogenic (PM2+PM5+PP3+PP4).

The third novel mutation, c.711‐2A>G, changed a conserved AG sequence to GG. The mutation site affects the normal splicing of *MITF* pre‐mRNA, resulting in a truncated protein with abnormal function or activity. Prediction tools (ESEfinder, MaxEntScan, BDGP Splice Site Prediction by Neural Network, and NetGene2) predict that this variant abolishes the accept splice site in intron 8 and prevents the transcription of exon 8 (PVS1). The variant meets PS4: Autosomal dominant: ≥2 probands with variant, and the frequency is 0 in dbSNP, ExAC, 1000 Genomes Project, or gnomAD databases. The mutation c.711‐2A>G is considered to be pathogenic (PVS1+PS4).

The mutation c.328C>T in exon 3 is a nonsense variant that was reported by Bocángel et al., (2018); it introduced a stop codon in the TAD domain.

## 4 DISCUSSION

Sensorineural hearing loss, iris pigmentary abnormalities, and a freckled face are the most common findings in WS. Dystopia canthorum (W index <1.95) is a clinical characteristic of WS1 and WS3. There was no dystopia canthorum, upper limb abnormalities, or Hirschsprung disease in our subjects. Therefore, the clinical diagnosis of the study subjects was WS2. The *MITF*, *SNAI2*, and *SOX10* genes are related to WS2.2 An estimated 15% of WS2 patients are heterozygous for *MITF* mutations (Read & Newton, 1997). *MITF* participates in the mechanisms of five other pathogenic genes: *SOX10* activates the *MITF* promoter either alone or in cooperation with *PAX3* in vitro (Bondurand et al., 2000), the *EDN3*/*EDNRB* signaling pathway affects the transcription of *MITF*, and *MITF* regulates the expression of *SNAI2*. *MITF* protein regulates the growth and differentiation of melanocytes by activating melanocyte genes, such as tyrosinase (*TYR*) and tyrosinase‐related protein 1 (*TYRP1*; Hou et al., 2006). The absence or dysregulation of melanocytes can cause degeneration of the organ of Corti and affect the internal potential of the cochlea (Hai et al., 2017; Liu et al., 2016), which is an important link in hearing. Different mutations have different pathogenic mechanisms, such as binding and activating downstream genes, phosphorylation, nuclear localization, and haploinsufficiency (Pingault et al., 2010).

Exons 7 to 9, which encode the bHLH‐Zip domain, are highly conserved in vertebrates and invertebrates (Pingault et al., 2010). The bHLH‐Zip domain is involved in regulating DNA‐binding activity and activating the *TYR* promoter. A functional decline and loss of the bHLH‐Zip domain results in the haploinsufficiency of the MITF protein and pigment abnormalities (Smith et al., 2000; Takeda et al., 2000). A splice site mutation in exon 7 that results in a truncated protein is associated with WS2 (George et al., 2016). According to the Deafness Variant Database, p.Arg217Gly, p.Arg217del, and p.Arg217Ile can lead to WS2. The exon 7 variant c.650G>A was found in the patient from family 3, resulting in the transformation of p.Arg217Lys. We speculate that amino acid 217 has a vital role in the function of the bHLH‐Zip domain. The heterozy gous mutation c.831dupC in exon 8, a frameshift variant, was detected in a 22‐year‐old male from family 1. Neither his parents nor his brother carries the mutation. The mutation leads to changes in 12 amino acids in the bHLH‐Zip domain. In two previous studies, nonsense mutations of exon 8 in the bHLH‐Zip domain were reported to cause sensorineural hearing loss, heterochromia iridis, and a freckled face (Bocángel et al., 2018; Morell et al., 1997) similar to our patients. The proband in family 4 had a novel heterozygous mutation (*MITF* c.711‐2A>G) in the splicing site, which results in a truncated protein. We believe that this mutation was pathogenic. A literature review suggested that truncated proteins or mutations disrupting dimerization result in WS2 via haploinsufficiency (Nobukuni et al., 1996). We hypothesized that the truncated protein caused by the novel mutation c.711‐2A>G eliminates the bHLH‐Zip structure.

Bocángel et al. reported the mutation c.328C>T in a Brazilian and the proband had sensorineural hearing loss, heterochromia iridis, and a freckled face (Bocángel et al., 2018) similar to our patients. The mutation c.328C>T in exon 3 is a nonsense variant that introduces a stop codon in the TAD domain, which may affect *MITF* transcriptional activity (Hartman & Czyz, 2015).

WS caused by *PAX3*, *EDNRB*, *EDN3*, or *SOX10* is characterized by intra‐ and interfamilial phenotypic variability and incomplete penetrance (Somashekar et al., 2019). Heterozygous variants in *MITF* also show incomplete penetrance in WS families (Yan et al., 2011). However, incomplete penetrance of *MITF* in WS2 is rare, first reported by Alehabib et al., (2020) Our patients in family 4 carried the same *MITF* mutation, but had different symptoms. We postulate that different genetic backgrounds, environmental factors, stochastic events, modifying genes, and epigenetics play a role in the penetrance of hearing loss (Pingault et al., 2010). The final appearance may be the result of complex interactions of many factors.

## 5 CONCLUSION

The patients’ clinical diagnoses were based on detailed clinical manifestations and the involved families also had genetic diagnoses. Three novel mutations and one recurrent mutation were identified in the *MITF* gene, which adds to the human gene mutation database and may be helpful for the prenatal genetic diagnosis of WS. Our findings provide novel insight into the mechanism underlying WS and the treatment of patients with WS.

## CONFLICT OF INTEREST

The authors declare no competing interests.

## 6 DATA AVAILABILITY STATEMENT

The generated data from this study are available from the corresponding authors upon a justifiable request.

## AUTHORS CONTRIBUTIONS

J. W., J. C., H. Y., and Y. Z. were involved in the study design. X. Y., Y. L., T. S., L. L., and Y. R. prepared the samples for the experiments. B. T. and W. X. carried out data analysis. J. W. drafted the manuscript. All the authors contributed to critical discussions and approved the final version of the manuscript.

## Supporting information

Table S1
