# Supplementary material for: Identification of novel MITF mutations in Chinese families with Waardenburg syndrome type II
Source: Mol Genet Genomic Med. 2021 Jul 29;9(9):e1770. doi: 10.1002/mgg3.1770 (PMC8457691; doi:10.1002/mgg3.1770)
Supplement: Supplementary file 1 — Table S1 [file MGG3-9-e1770-s001.doc]

Supplementary materials

| Gene | primer sequences (forward) | primer sequences(reverse) |
| --- | --- | --- |
| *MITF:* c.328C>T | 5’‑CATCTTGTTGCTGTGCCATC-3’ | 5’‑AAGGTGTGATCCACCACAAA-3 |
| *MITF:* c.650G>A | 5’‑CGTTGTCATGACCTGGAGAA-3’ | 5’‑CAAAGGGAGAGGGGAGACTT-3’ |
| *MITF:* c.711‑2A>G | 5’‑CTTATCCATGTAACCAAGCA-3’ | 5’‑CACACACACAGAATCCACAAA-3’ |
| *MITF:*c.831dupC | 5’‑TCAAAAAGAAATGGAGTGCTTTGA-3’ | 5’‑TCAACTCCCCTATGGCTCATTA-3 |
